# Tau deletion promotes brain insulin resistance

**DOI:** 10.1084/jem.20161731

**Published:** 2017-08-07

**Authors:** Elodie Marciniak, Antoine Leboucher, Emilie Caron, Tariq Ahmed, Anne Tailleux, Julie Dumont, Tarik Issad, Ellen Gerhardt, Patrick Pagesy, Margaux Vileno, Clément Bournonville, Malika Hamdane, Kadiombo Bantubungi, Steve Lancel, Dominique Demeyer, Sabiha Eddarkaoui, Emmanuelle Vallez, Didier Vieau, Sandrine Humez, Emilie Faivre, Benjamin Grenier-Boley, Tiago F. Outeiro, Bart Staels, Philippe Amouyel, Detlef Balschun, Luc Buee, David Blum

**Affiliations:** 1 Université de Lille, Institut National de la Santé et de la Recherche Medicale (INSERM), CHU Lille, UMR-S 1172 JPArc, Lille, France; 2 LabEx DISTALZ (Development of Innovative Strategies for a Transdisciplinary approach to ALZheimer’s disease), Lille, France; 3 Laboratory of Biological Psychology, Faculty of Psychology and Educational Sciences, KU Leuven, Leuven, Belgium; 4 Université de Lille, INSERM, CHU Lille, Institut Pasteur de Lille, U1011EGID, Lille, France; 5 Université de Lille, INSERM, CHU Lille, Institut Pasteur de Lille, U1167 RID-AGE Facteurs de Risque et Déterminants Moléculaires des Maladies Liées au Vieillissement, Lille, France; 6 INSERM U1016, CNRS UMR8104, Université Paris Descartes Sorbonne Paris Cité, Institut Cochin, Paris, France; 7 Department of Experimental Neurodegeneration, Center for Nanoscale Microscopy and Molecular Physiology of the Brain, University Medical Center Goettingen, Goettingen, Germany; 8 Neurological Disorders Research Center, Qatar Biomedical Research Institute, Hamad Bin Khalifa University, Doha, Qatar

## Abstract

The molecular pathways underlying tau pathology–induced synaptic/cognitive deficits and neurodegeneration are poorly understood. One prevalent hypothesis is that hyperphosphorylation, misfolding, and fibrillization of tau impair synaptic plasticity and cause degeneration. However, tau pathology may also result in the loss of specific physiological tau functions, which are largely unknown but could contribute to neuronal dysfunction. In the present study, we uncovered a novel function of tau in its ability to regulate brain insulin signaling. We found that tau deletion leads to an impaired hippocampal response to insulin, caused by altered IRS-1 and PTEN (phosphatase and tensin homologue on chromosome 10) activities. Our data also demonstrate that tau knockout mice exhibit an impaired hypothalamic anorexigenic effect of insulin that is associated with energy metabolism alterations. Consistently, we found that tau haplotypes are associated with glycemic traits in humans. The present data have far-reaching clinical implications and raise the hypothesis that pathophysiological tau loss-of-function favors brain insulin resistance, which is instrumental for cognitive and metabolic impairments in Alzheimer’s disease patients.

## Abstract (precis)

Physiological functions of tau remain ill defined. In the present study, Marciniak et al. uncover a novel function of tau in its ability to regulate brain insulin signaling and discuss the pathophysiological implications of these findings for Alzheimer’s disease and tauopathies.

## Introduction

Alzheimer’s disease (AD) is a neurodegenerative disorder characterized by the progressive development of memory deficits. AD is neuropathologically defined by extracellular accumulation of amyloid-β peptides into amyloid plaques and intraneuronal fibrillar aggregates of hyper- and abnormally phosphorylated tau proteins (Masters et al., 1985; Sergeant et al., 2008). Tau pathology is observed early in the brainstem and entorhinal cortex (Braak et al., 2011), and its progression in the cortex from entorhinal cortex to hippocampus to neocortex corresponds to the progression of AD symptoms (Duyckaerts et al., 1997; Grober et al., 1999; Jucker and Walker, 2013).

Pathways underlying tau pathology–induced synaptic/cognitive deficits and neurodegeneration are not well understood. The prevalent hypothesis is that hyperphosphorylation, misfolding, and fibrillization of tau (“tau pathology”) impair synaptic plasticity and cause degeneration (Wang and Mandelkow, 2016), with a large agreement toward a toxic gain of function. However, we cannot rule out that tau pathology may also result in the loss of specific physiological tau functions that finally contribute to neuronal dysfunction, but this hypothesis has been poorly investigated (Trojanowski and Lee, 2005; Morris et al., 2011). Constitutive deletion of tau does not lead to lethality or neurodegeneration (Morris et al., 2011; Wang and Mandelkow, 2016), presumably because of compensatory mechanisms (Harada et al., 1994). However, tau is obviously needed for normal brain function, because tau deletion has been associated with brain iron accumulation (Lei et al., 2012) and deficits in synaptic plasticity and cognition (Kimura et al., 2014; Ma et al., 2014; Ahmed et al., 2015).

There is limited knowledge on the physiological roles of tau. The most extensively described neuronal functions of tau are related to its ability to bind microtubules, to regulate their assembly and spacing, as well as to control axonal transport (Dixit et al., 2008; Sergeant et al., 2008; Morris et al., 2011; Wang and Mandelkow, 2016; Lacovich et al., 2017). Recently, other functions have emerged, such as the ability of tau to control neuronal excitability at dendritic spines (Ittner et al., 2010) or protect nucleic acids from oxidative stress (Sultan et al., 2011; Violet et al., 2014). However, as a scaffold protein (Arendt et al., 2016), broader tau functions are expected, and thus, better knowledge of tau functions is essential to gain insight into the pathophysiological processes underlying AD and tauopathies. In the present study, we uncover a novel function of tau in its ability to regulate brain insulin signaling and discuss the pathophysiological implications of these findings.

## Results and discussion

### Tau deletion impairs hippocampal response to insulin

**Figure 1.**
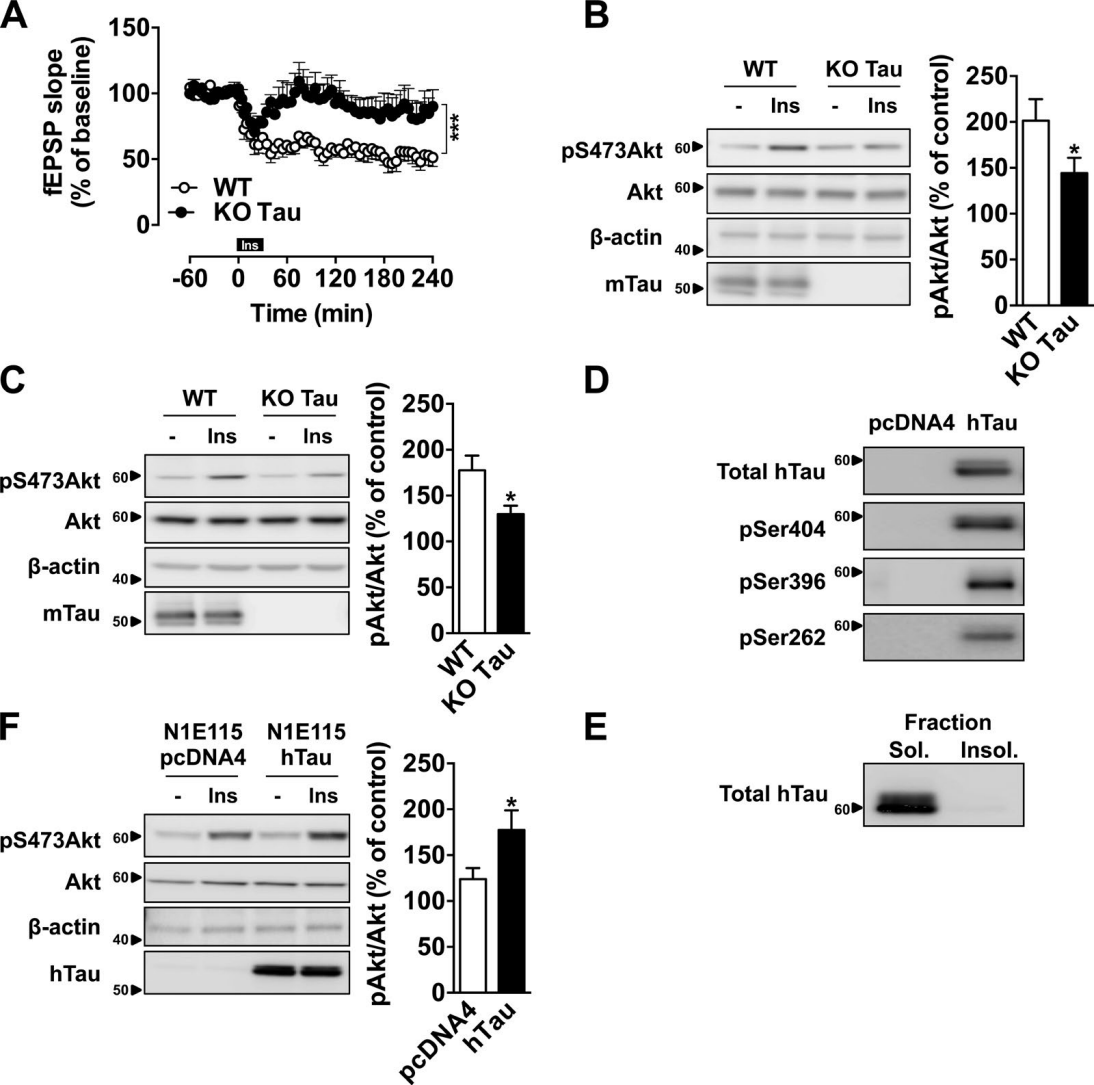
**Tau regulates hippocampal response to insulin.** (A) Hippocampal LTD induced by 1 µM insulin (30 min) in tau KO and WT mice. Each point represents mean ± SEM normalized to baseline values preceding the application of insulin (***, P < 0.001, two-way ANOVA). (B) Akt phosphorylation in hippocampal slices from tau KO mice and littermate controls, 10 min after 200 nM insulin treatment (*, P < 0.05, Student’s *t* test). (C) Akt phosphorylation in the hippocampus of tau KO mice and littermate controls, 1h after an icv injection of 2 µl at 5 mg/ml insulin (*, P < 0.05, Student’s *t* test). Tau KO mice were 7–12 mo old. (D) hTau expression and phosphorylation and (E) hTau detection in sarkosyl-soluble and -insoluble fractions from N1E115 cells overexpressing WT 1N4R human tau isoform. (F) Akt phosphorylation in N1E115 overexpressing WT hTau or transfected by empty vector, 10 min after insulin treatment at 200 nM (*, P < 0.05, Student’s *t* test). Controls are indicated as open circles/bars and tau KO/overexpressing cells as black circles/bars. Data in A–C show mean ± SEM from six (A), six to eight (B), and six or seven (C) mice per group from six (A), two (B), or three (C) independent experiments. Data in D and E show results from one independent experiment. Data in F show mean ± SEM from eight wells per group from five independent experiments. Ins, insulin. Molecular mass is indicated in kilodaltons.

Tau deletion impairs hippocampal synaptic plasticity and memory in mice (Kimura et al., 2014; Ma et al., 2014; Ahmed et al., 2015), but the underlying mechanisms remain unclear. Here, we investigated a potential interaction between tau and hippocampal insulin signaling, which is known to regulate hippocampal plasticity and reference memory (Fernandez and Torres-Alemán, 2012; De Felice and Benedict, 2015; Grillo et al., 2015). We first addressed the impact of tau deletion upon hippocampal responsiveness to insulin using hippocampal slices from tau KO mice (Tucker et al., 2001). We evaluated insulin-induced hippocampal long-term depression (LTD; Van Der Heide et al., 2005) by taking advantage of an induction protocol, which uses a 30-min incubation with 1 µM insulin to evoke LTD of extracellular field excitatory postsynaptic potentials (fEPSPs). As shown in Fig. 1 A, in WT mice, insulin induced a marked decay of recordings (47.9 ± 7.2%) compared with baseline (240 min; P = 0.0011 vs. baseline). Interestingly, in tau KO mice, the magnitude of hippocampal LTD was significantly reduced compared with littermate controls (240 min, slope at 87.5 ± 12.1% of baseline, 1–240 min, P < 0.001 vs. WT, two-way ANOVA; Fig. 1 A). Impaired insulin responsiveness was confirmed in another set of experiments that revealed a significant reduction of hippocampal Akt ex-vivo phosphorylation, after insulin application to slices (Fig. 1 B), but also in vivo, after intracerebroventricular (icv) injection of the hormone (Fig. 1 C). These data support that tau deletion leads to hippocampal insulin resistance. To test whether tau overexpression was, conversely, associated with improved response to insulin, we used a model of N1E115 neuroblastoma cells that overexpress WT 1N4R human tau (Fig. 1 D), in the absence of tau aggregation (Fig. 1 E). In this model, we observed a higher responsiveness to insulin treatment in neuroblastoma N1E115 cells overexpressing hTau than in controls (Fig. 1 F). Altogether, the present data support the ability of tau to modulate hippocampal responsiveness to insulin and open the possibility that tau itself regulates insulin signaling.

### Interaction between tau and insulin signaling

**Figure 2.**
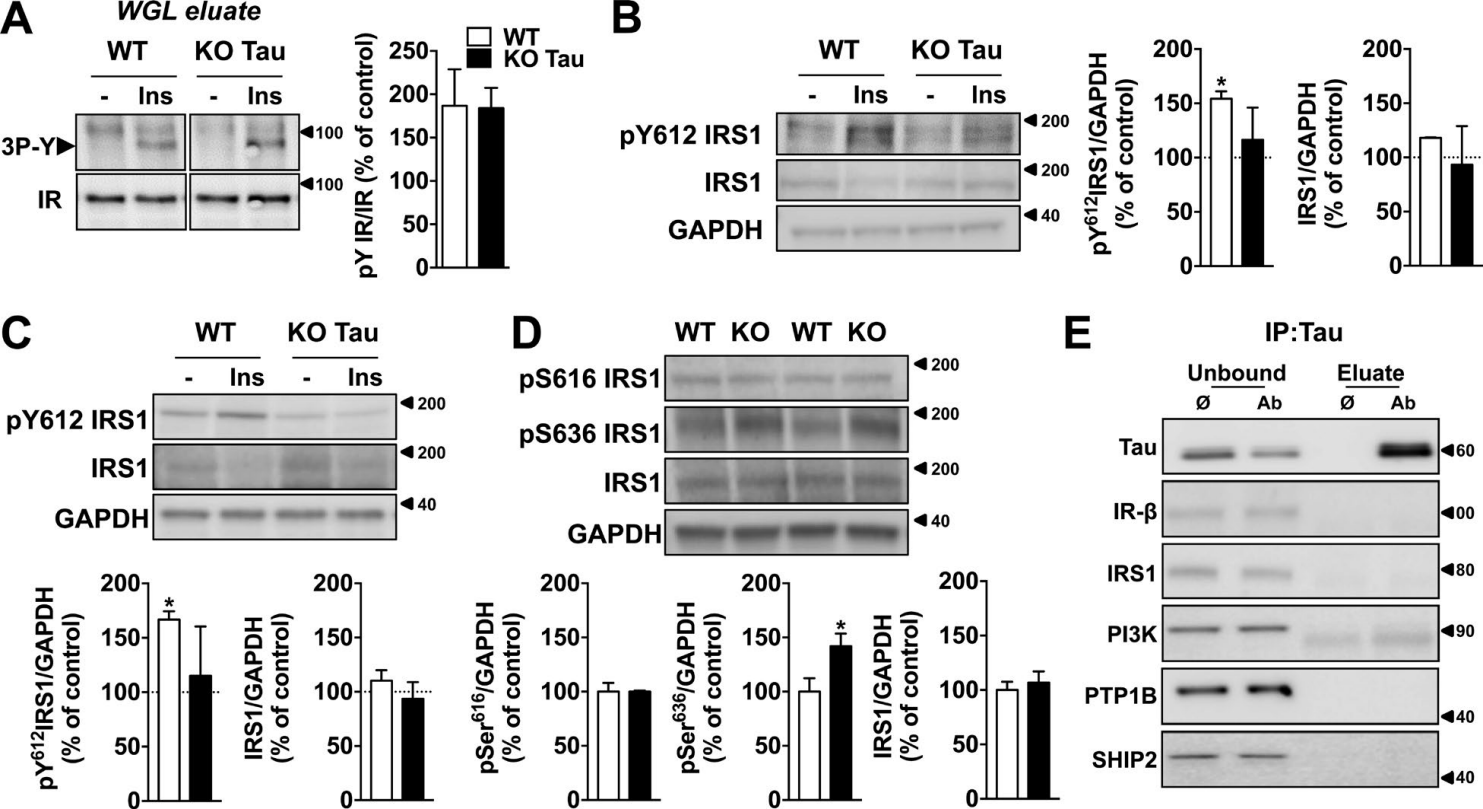
**Impact of tau deletion upon IR and IRS-1.** (A) IRs from hippocampal slice extracts (400 µg protein) were precipitated on wheat-germ lectin (WGL) agarose beads, eluted with Laemmli sample buffer, and submitted to SDS-PAGE followed by Western blotting. Insulin-induced phosphorylation of the IR on the three tyrosines of the kinase domain was evaluated using anti–IRpY1158-1162-1163 antibody. The amount of IR present in each track was evaluated by reprobing the membrane with an anti-IRβ antibody. (B) Tyrosine phosphorylation of IRS-1 in hippocampal slices from tau KO mice and littermate controls 10 min after 200 nM insulin treatment (*, P < 0.05, Student’s *t* test). Total IRS-1 remains constant. (C) Tyrosine phosphorylation of IRS-1 in the hippocampus of KO tau mice and littermate controls 1 h after icv injection of 2 µl insulin at 5 mg/ml (*, P < 0.05, Student’s *t* test). Total IRS-1 remains constant. (D) Serine phosphorylation of IRS-1 in tau KO mice and littermate controls. (*, P < 0.05, Student's *t* test). (E) Coimmunoprecipitation experiments between hTau and different members of the insulin-signaling pathway in N1E115 cells transfected with WT 1N4R hTau. Tau interacted with PTEN only (Fig. 3 A). Ø indicates negative control of immunoprecipitation (IP; without primary antibody; only mouse anti-IgG beads); Ab indicates immunoprecipitation with the primary antibody and mouse anti-IgG beads. Unbound indicates the immunoprecipitation supernatant samples, whereas eluate indicates the immunoprecipitation complexes. Controls are indicated as open bars and tau KO animals as black bars. KO mice were 7–12 mo old. Data in A–D show mean ± SEM from four (A), three (B), three (C), and five (D) mice per group from two independent experiments. Coimmunoprecipitations were obtained from at least two independent experiments. Molecular mass is indicated in kilodaltons.

Next, we aimed to understand the mechanisms underlying the ability of tau to modify the response to insulin. In a first attempt, we evaluated whether insulin-induced tyrosine phosphorylation of insulin receptor (IR) could be modified after tau deletion. Compared with respective littermate controls, tyrosine phosphorylation of IRs remained unaffected by tau deletion after insulin application to slices (Fig. 2 A). Further, IR membrane expression remained unaffected by hTau overexpression in neuroblastoma cells (not depicted).

Next, we investigated the impact of tau deletion upon IRS-1, which is an important node of insulin signaling. Tyrosine phosphorylation of IRS-1 by IR leads to the downstream activation of Akt, whereas the phosphorylation of IRS-1 on multiple serine residues inhibits IRS-1 activity, leading to insulin resistance (Copps and White, 2012). Several works notably associated abnormal IRS-1 serine phosphorylation to brain insulin resistance in the context of AD and tauopathies (Ma et al., 2009; Bomfim et al., 2012; Talbot et al., 2012; Yarchoan et al., 2014). Here, we found that insulin-induced hippocampal tyrosine phosphorylation of IRS-1 was reduced in tau KO mice after slice treatment (Fig. 2 B) or after icv injection with the hormone (Fig.2 C). In line with an impairment of IRS-1 activity, we also observed that serine phosphorylation at Ser636 was increased in the hippocampus of tau KO animals (Fig. 2 D). Serine phosphorylation of IRS-1 has been suggested to be related to a bottom-up (rather than top-down) effect involving downstream kinases such as JNK (Ma et al., 2009). This fits well with the previously demonstrated JNK activation in tau KO mice (Ma et al., 2014). This is also in line with the idea that phosphorylated serines/threonines of IRS-1 could inhibit its tyrosine phosphorylation (Mothe and Van Obberghen, 1996). Together, the present data thus support that tau deletion is associated with impaired IRS-1 function. Tau KO mice exhibit changes reminiscent of the AD brain; that is, a concomitant reduction of insulin-induced IRS-1 tyrosine phosphorylation (Talbot et al., 2012) with an increased IRS-1 phosphorylation at Ser636 (Bomfim et al., 2012).

**Figure 3.**
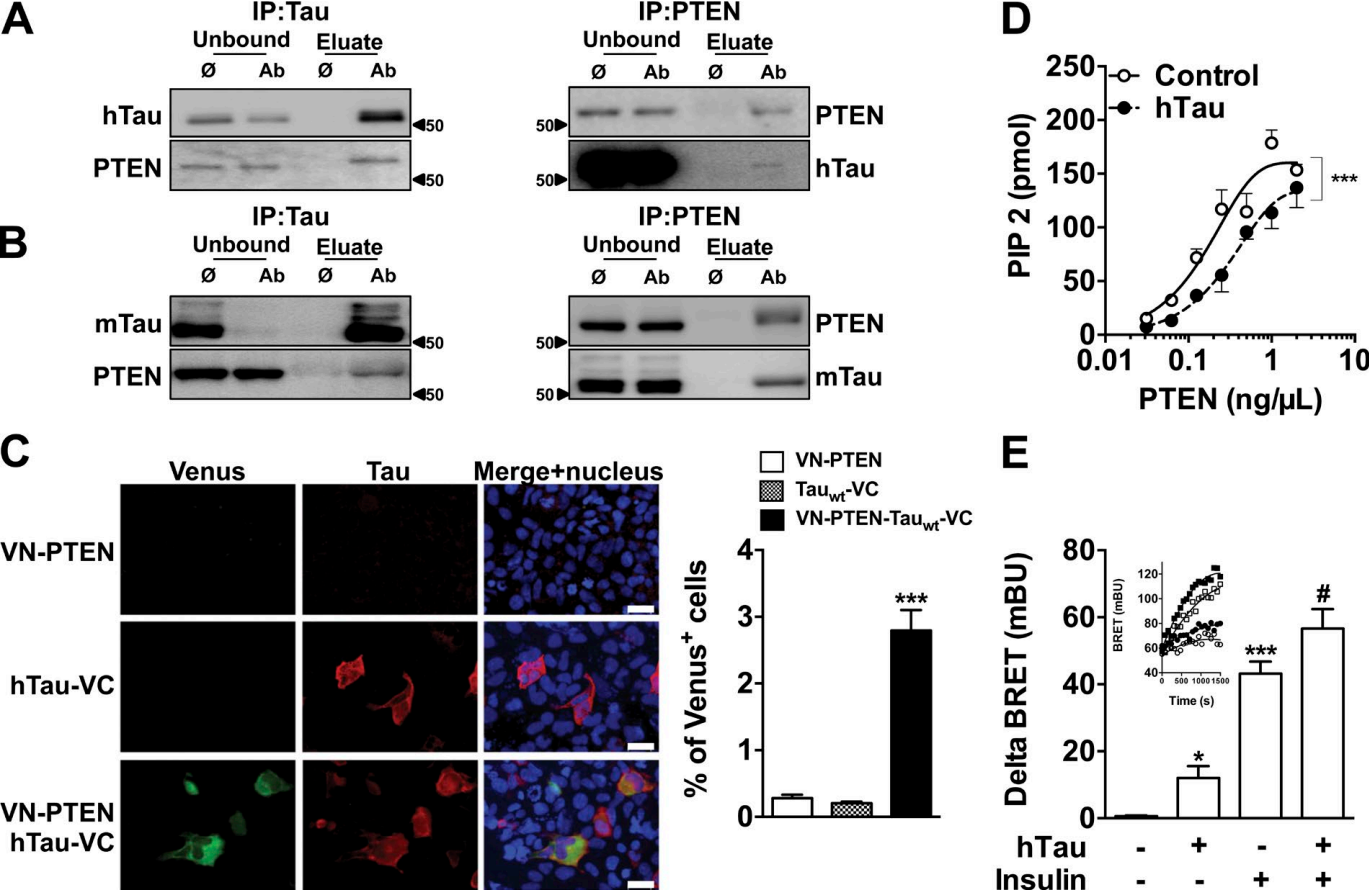
**Tau interacts with PTEN and modulates its lipid phosphatase activity.** (A and B) Coimmunoprecipitation of hTau (WT 1NR4) overexpressed in N1E115 cell (A) and mouse tau in the hippocampus of WT mice (B) with PTEN. Data are representative of two independent experiments from independent samples. (C) Bimolecular fluorescence complementation (BiFC) assay confirms the interaction between WT 1N4R tau and PTEN in the cytoplasm of HEK-293 cells. Data are representative of three independent experiments (***, P < 0.001 vs. VN-PTEN; one-way ANOVA, LSD Fisher’s post-hoc test). Bars, 30 μm. (D) In cell-free conditions, lipid phosphatase activity of PTEN is decreased in the presence of recombinant hTau protein (control, open circles; WT 1N4R, black circles; ***, P < 0.001, two-way ANOVA). Note that PTEN activity is not modulated in the presence of an irrelevant protein (albumin; not depicted). Data show mean ± SEM from six to eight assays per condition from three independent experiments. (E) Modulation of PtdIns(3,4,5)P3 production by hTau (WT 1N4R) using BRET. In each experiment, delta BRET was determined by removing mean basal BRET values from mean BRET values obtained for each experimental condition (20 BRET measurements per experimental condition). Inset: typical BRET experiment showing real-time insulin effect on PIP3 production is presented (open circle, control/vehicle; closed circles, hTau/vehicle; open square, control/insulin; closed square, hTau/insulin; *, P < 0.05; ***, P < 0.001 vs. control; #, P < 0.05 vs. insulin condition; one-way ANOVA, LSD Fisher’s post-hoc test). Data show mean ± SEM from five assays per condition from two independent experiments. Molecular mass is indicated in kilodaltons.

Further, we performed coimmunoprecipitation experiments in neuroblastoma N1E115 cells expressing WT hTau to evaluate whether tau interacts with elements controlling insulin signaling (Fig. 2 D). Tau did not interact with IR or IRS-1 (Fig. 2 E). Although previous biochemical studies suggested that tau may bind to the p85α subunit of phosphoinositide 3-kinase (PI3K; Reynolds et al., 2008), we did not observe coimmunoprecipitation of PI3K (p85) with tau (Fig. 2 E). As our results indicate that tau deletion blunts the response to insulin, we also addressed a possible interaction of tau with three phosphatases known to inhibit insulin signaling: protein tyrosine phosphatase 1B (PTP1B), SH2 domain containing inositol 5-phosphatase 2 (SHIP2), and phosphatase and tensin homologue on chromosome 10 (PTEN). Although tau did not interact with PTP1B and SHIP2 (Fig. 2 E), we found it coimmunoprecipitated with PTEN in WT hTau-transfected N1E115 cells (Fig. 3 A) and also in mouse brain tissue (Fig. 3 B). A direct interaction between WT hTau and PTEN was further confirmed using the bimolecular fluorescence complementation (BiFC) assay (Fig. 3 C). PTEN is known as a negative regulator of the PI3K-Akt pathway and, using in vitro and bioluminescence resonance energy transfer (BRET) experiments, we found that PTEN activity and cellular PtdIns(3,4,5)P3 production were dependent on tau. First, we demonstrated that recombinant WT hTau impaired the lipid phosphatase activity of recombinant PTEN by decreasing PtdIns(3,4,5)P3 dephosphorylation into PtdIns(4,5)P2 in cell-free conditions (Fig. 3 D). In addition, using an in cellulo BRET assay (Pierre-Eugene et al., 2012), we measured that expression of tau not only was sufficient to favor PtdIns(3,4,5)P3 formation in N1E115 cells by itself but also potentiated the enhancing effects of insulin on PtdIns(3,4,5)P3 formation (Fig. 3 E). Altogether, the present data unravel the ability of tau to reduce PTEN activity and to favor PtdIns(3,4,5)P3 production, explaining, apart the aforementioned effect toward IRS-1, the reduced insulin responsiveness seen in conditions where tau is deleted (Fig. 1; Ortega-Molina and Serrano, 2013). Therefore, our data indicate that interaction between tau and PTEN restrains PTEN’s lipid phosphatase activity, favoring pro-cognitive insulin signaling. Interestingly, PTEN has been shown to contribute instrumentally to the memory alterations in mouse models of AD (Knafo et al., 2016), supporting a critical role of this phosphatase in the regulation of hippocampal synaptic plasticity. However, the precise identification and characterization of tau functions, whose loss leads to insulin resistance in AD, is left to further research.

### Tau deletion inhibits anorexigenic effect of brain insulin administration and leads to metabolic disturbances

**Figure 4.**
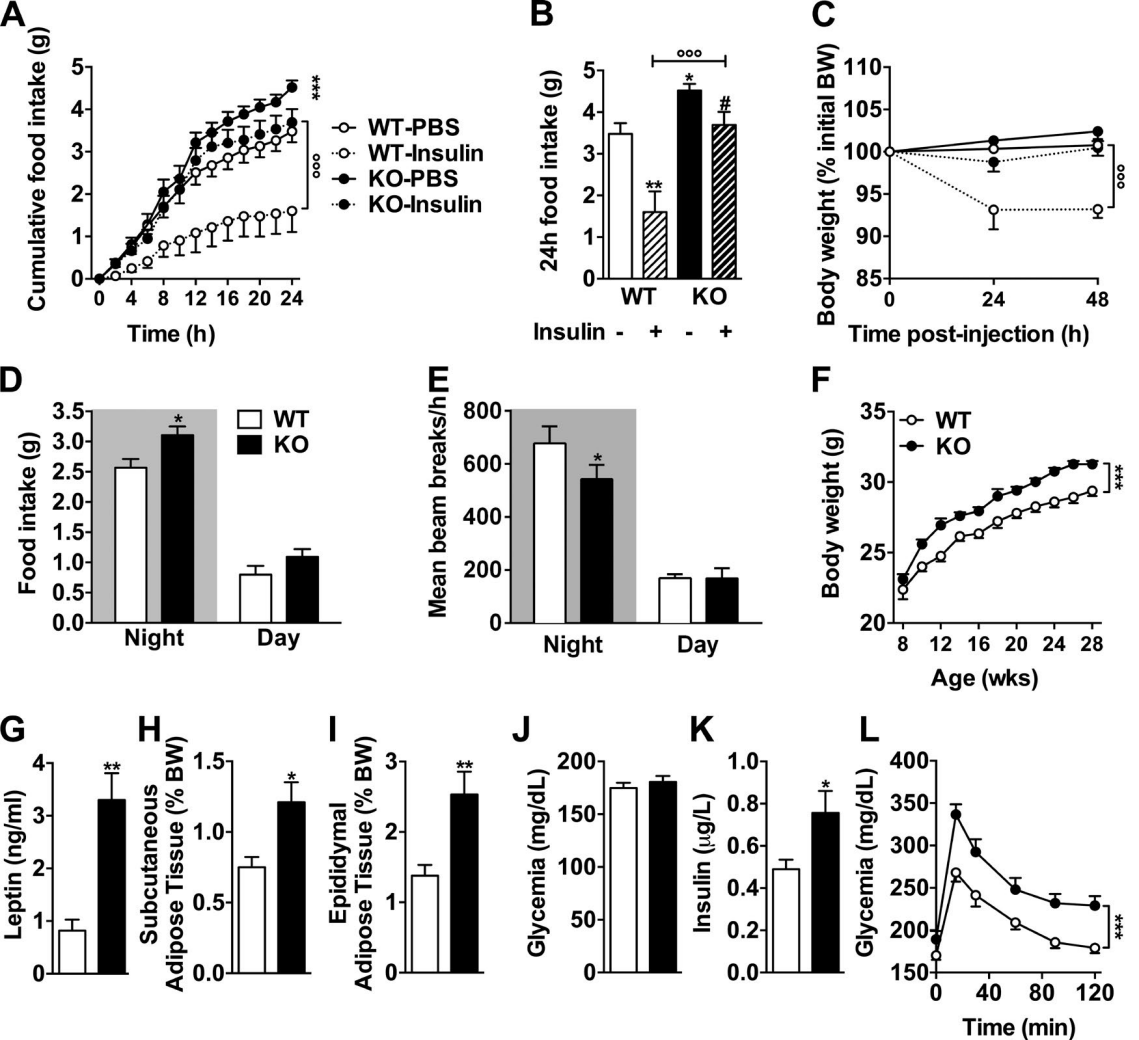
**Tau deficiency inhibits an anorexigenic effect of brain insulin administration leading to metabolic disturbances.** (A) Cumulative food intake for 24 h measured using metabolic cages in tau KO and littermate controls intracerebroventricularly injected first with vehicle and then 2 µl insulin (5 mg/ml; ***, P < 0.001 vs. WT-PBS; °°°, P < 0.001 vs. tau KO/insulin; two-way ANOVA). (B) Cumulated food intake in WT and tau KO mice 24 h after vehicle or insulin brain injection (same animals as in A; *, P < 0.05; **, P < 0.01 vs. WT-PBS; #, P < 0.05 vs. WT/insulin; °°°, P < 0.001 vs. tau KO/insulin; one-way ANOVA, LSD Fisher’s post-hoc test). (C) 48-h body weight variation after vehicle or insulin brain injection in tau KO and WT littermates (same animals as in A; °°°, P < 0.001 vs. tau KO/insulin; two-way ANOVA). Data in A–C show mean ± SEM from 5–10 mice per group from three independent experiments. (D) Mean food intake (*, P < 0.05, two-way ANOVA). (E) Ambulatory activity (*, P < 0.05, two-way ANOVA). (F) Body weight gain (***, P < 0.05, two-way ANOVA). (G) Plasma leptin levels (**, P < 0.01, Student’s *t* test). (H and I) Adipose tissue weight (*, P < 0.05; **, P < 0.01, Student’s *t* test). (J) Glycemia. (K) Insulinemia (*, P < 0.05, Student’s *t* test). (L) Intraperitoneal glucose tolerance test (***, P < 0.05, two-way ANOVA) in tau KO mice and littermate controls. Quantifications represent mean ± SEM. Controls are indicated as open circles/bars, tau KO as black circles/bars. Dashed lines/bars represent insulin-treated animals. Mice were 6–8 mo old at time of experiments and sacrifice. Metabolic data in C–L show mean ± SEM from 10–12 (D), 9–12 (E), 13–27 (F), 4–5 (G), 11–14 (H and I), 15–17 (J), 20–23 (K), and 12–14 (L) mice per group acquired from three independent experiments.

Considering the broad functions of insulin signaling in the adult brain (Fernandez and Torres-Alemán, 2012), we assumed that its regulation by the pan-neuronal tau protein might not be restricted to hippocampus. Thus, we next asked whether constitutive tau deletion in mice would modulate the known ability of intracerebroventricularly administered insulin (bilateral icv injection in lateral ventricle) to reduce food intake and body weight gain (Woods et al., 1979; Brown et al., 2006). As expected, 24-h food intake was significantly reduced in WT littermate animals after icv insulin administration (Fig. 4 A). Strikingly, the anorexigenic effect of icv insulin was strongly reduced in tau KO mice (Fig. 4, A and B). Accordingly, icv insulin was able to promote body weight loss in WT, but not tau KO, mice (Fig. 4 C). These data indicated that brain insulin signaling is blunted in tau KO mice. Impaired insulin response was not associated with impaired response to leptin. Indeed, as expected, 3-d intraperitoneal leptin injections reduced food intake and body weight in WT littermate animals by 27.3 ± 6.6% (four replicates, P = 0.02) and 3.8 ± 0.4% (four replicated, P = 0.002), respectively. Similar changes were observed in tau KO animals (−21.3 ± 6.4% and −3.8 ± 0.5%, four replicates), suggesting that leptin sensitivity remained unaffected by tau deletion.

Both genetic deletion of neuronal IRs and hypothalamic IR knockdown have been previously shown to increase food intake, body weight gain, and adiposity in rodents (Brüning et al., 2000; Obici et al., 2002; Grillo et al., 2007). In accordance with impaired insulin signaling in the brain of tau KO mice, we found that the latter exhibited enhanced food intake when fed ad libitum (Fig. 4, A and D) and body weight gain when compared with WT littermates (Fig. 4 F) in the absence of a change in body weight at weaning (Fig. S1 A). Adiposity was increased in tau KO mice, as exemplified by enhanced circulating leptin (Fig. 4 G) and adipose tissue weight (Fig. 4, H and I). Furthermore, tau deletion was also associated with a significant hyperinsulinemia (Fig. 4 K) and glucose intolerance (Fig. 4 L). As shown in Fig. S2, tau heterozygous mice, whose brain tau expression was reduced when compared with WT littermates (Fig. S2 I), exhibited a metabolic phenotype in-between WT and tau KO mice, particularly regarding food intake and glucose tolerance (Fig. S2, A–H). When combined, these data indicate that the neuronal protein tau plays an important role in the regulation of energy homeostasis and peripheral metabolism. The metabolic changes seen in tau KO mice are likely caused by the inability of the hypothalamus to properly respond to insulin. However, the contribution of peripheral tau to the metabolic changes observed is also possible. Indeed, tau mRNA expression has been found in normal human exocrine pancreas (Neuville et al., 1995), and tau protein has been shown to colocalize with insulin-positive β-cells at the islets of Langerhans of pancreatic tissue from tumor resections (Maj et al., 2010). Further, expression of tau in insulinoma cells transiently reduces insulin secretion, whereas expression or knockdown of tau in rat insulinoma cells leads to increased or decreased insulin transcription in the same cells, respectively (Maj et al., 2016). Therefore, these data support a role of tau in the regulation of insulin transcription and secretion by insulinoma cancer cells (although this has not been demonstrated in primary β-cells). We thus cannot rule out that the effect of tau deletion upon insulin levels or glucose tolerance is the consequence of pancreatic regulations. Interestingly, increased body weight gain and adiposity of tau KO mice (Fig. 4, F, H, and I) was also associated with a reduced ambulatory activity. The relationship between insulin signaling and motor function has been poorly documented in the literature. However, Mysoet et al. (2017) have recently shown that the cortical administration of IGF-1, a protein that is structurally similar to insulin and shares a similar signaling pathway, induces an increase in motor activity of rats. Other recent data point to a correlation between insulin resistance and disease severity in spinal and bulbar muscular atrophy (Nakatsuji et al., 2017), supporting a role of peripheral metabolic alterations and muscle function. It is thus possible that defective insulin signaling in tau KO mice leads to impaired motor activity. This is in line with previous data (Lei et al., 2012) but remains a matter of debate (Morris et al., 2013).

### Tau haplotypes are associated with glycemic traits in humans

To address whether tau impacts peripheral metabolism in humans, we explored the potential association between microtubule-associated protein *tau* (MAPT; tau) haplotypes and metabolic traits using published genome-wide association study (GWAS) data. Although the precise impact remains subject to debate, MAPT haplotypes are thought to be associated with functional changes in the tau protein (Trabzuni et al., 2012). Meta-analysis of 21 GWASs indicated that the MAPT H1/H2 tagging single-nucleotide polymorphism (SNP) rs1052553 was not associated with fasting glucose (P = 0.42) or fasting insulin (P = 0.79; 46,186 nondiabetic participants; Table 1; Dupuis et al., 2010). However, among GWASs with oral glucose tolerance test results, the rs1052553 A allele (which captures the H1 haplotype) was associated with a higher 2-h glucose level (β = 0.096 mmol/L, P = 0.00006, *n* = 15,234 nondiabetic participants; Saxena et al., 2010) and a lower 30-min insulin secretion (β = −0.063 pmol/L, P = 0.035, *n* = 5,318 nondiabetic participants; Prokopenko et al., 2014). These data are in agreement with glucose intolerance observed in heterozygous and tau KO mice (Fig. 4 L and Fig. S2 G). In line with a metabolic role for tau, a previous GWAS found a significant association between the locus 17q21.31 containing *MAPT* and food addiction (Cornelis et al., 2016).

**Table 1.** GWAS meta-analysis assessing the impact of MAPT haplotypes on glycemic traits in European individuals

Variable	N	Beta	SE	P	Reference
Fasting glucose (mmol/liter)	46,186	0.004	0.005	0.42	Dupuis et al., 2010
Fasting insulin (pmol/liter)	46,186	−0.001	0.005	0.79	Dupuis et al., 2010
2-h glucose (after OGTT) adjusted for BMI (mmol/liter)	15,234	0.096	0.024	5.9E-05	Saxena et al., 2010
30-min insulin secretion (after OGTT, ln) adjusted for BMI (pmol/liter)	5,318	−0.063	0.030	0.035	Prokopenko et al., 2014

Data were downloaded from www.magicinvestigators.org. Specific regression coefficients (beta) and standard error (SE) represent changes in the variable per copy of the rs1052553 A major allele (H1 haplotype, frequency = 0.79). Trait values for fasting insulin and insulin secretion 30 min after an oral glucose tolerance test (OGTT) were naturally log transformed (ln). Results are adjusted for age, sex, study-specific covariates, and body mass index (BMI) where indicated. N, sample size.

### Concluding remarks

Physiological functions of tau are largely unknown. The present data demonstrate a new role for tau in its ability to regulate brain insulin signaling (Fig. S3). Our data particularly indicate that tau deletion not only impairs insulin-induced hippocampal plasticity but also blunts the anorexigenic effect of this hormone. These observations are particularly interesting in the physiopathological context of AD and tauopathies. Indeed, brain insulin resistance is a cardinal feature of the AD brain (Moloney et al., 2010; Liu et al., 2011; de la Monte, 2014; Stanley et al., 2016a). Postmortem brains from AD patients exhibit a reduced responsiveness to insulin, which is correlated with memory deficits (Talbot et al., 2012). Although a few studies support the involvement of Aβ oligomers as a potential trigger for neuronal insulin resistance (Zhao et al., 2008; De Felice, 2013; Clarke et al., 2015; but see the results recently discussed by Stanley et al., 2016b), the role of tau remained completely unknown. Interestingly, other data emphasize brain insulin resistance in postmortem brains of patients with tauopathies (Yarchoan et al., 2014), indeed opening to a presumable role for tau itself.

Pathways underlying tau pathology-induced neuronal dysfunctions are not well understood. Pathological tau species are generally seen as the trigger for synaptic and cognitive impairments (Wang and Mandelkow, 2016). Hippocampal insulin signaling is recognized to favor plasticity and memory (Fernandez and Torres-Alemán, 2012; De Felice and Benedict, 2015; Grillo et al., 2015), a concept evidenced in humans by the ability of intranasal administration of insulin to improve cognition of AD patients (Reger et al., 2008; Stanley et al., 2016a). By showing that tau deletion blunts hippocampal response to insulin, our data support the possibility that impaired tau function could also contribute to abnormal plasticity seen in AD and tauopathies. This is in line with the observations of defective synaptic regulation and cognition in tau KO mice (Kimura et al., 2014; Ma et al., 2014; Ahmed et al., 2015). Thus, phenotypes observed in the tau KO mice are reminiscent of dysfunctions observed in AD and related tauopathies. Altogether, our data further suggest that tau is more than a microtubule-associated protein. In fact, tau is a scaffold protein (Arendt et al., 2016) that has been involved in the signal transduction of Src-family tyrosine kinases (Ittner et al., 2010; Burnouf et al., 2013), cyclin-dependent kinases (Vincent et al., 1996; Hamdane et al., 2005), and insulin signaling. Thus, impaired physiological tau function is prone to impact on signal transduction in neurons and contribute to plasticity alterations.

Besides the hippocampus, our data indicate that regulation of insulin signaling by tau also involves the hypothalamus, a known brain target of insulin. Our experimental data demonstrate that the anorexigenic effect of insulin (Woods et al., 1979; Brown et al., 2006) is lost in tau KO mice, but combined with human genetic studies (Table 1), our data particularly show that tau regulates energy homeostasis and peripheral metabolism. The incidence of type 2 diabetes is higher in AD patients (Janson et al., 2004). Moreover, in line with the occurrence of hypothalamic dysfunction in AD (Ishii and Iadecola, 2015), affected individuals exhibit fasting hyperglycemia and hyperinsulinemia as well as impaired glucose metabolism (Craft et al., 1992, 1998; Meneilly and Hill, 1993; Razay and Wilcock, 1994; Ma et al., 2016). Therefore, brain insulin resistance might be a crucial pathological mechanism that interactively drives, and is part of, the progression causing the cognitive and metabolic dysfunctions seen in AD patients. Impact of peripheral metabolic disturbances on AD has been commonly evaluated and experimental data confirm the detrimental effects of obesity, insulin resistance, and type 2 diabetes (T2D) toward β-amyloid and tau lesions as well as associated cognitive impairments (Takeda et al., 2010; Mayeux and Stern, 2012; Papon et al., 2013). Our data support a mutual pathophysiological relationship. Indeed, tau deletion leads to the development of peripheral metabolic abnormalities, in line with metabolic impairments seen in AD patients (Craft et al., 1992; Meneilly and Hill, 1993; Razay and Wilcock, 1994; Turner et al., 2013). Impaired tau function could therefore contribute to metabolic changes seen in AD patients. Overall, our data support the existence of a detrimental vicious circle linking peripheral metabolism dysregulations and lesion development in AD.

Altogether, our data demonstrate a novel physiological function of tau protein in the control of brain insulin signaling that could contribute to cognitive and metabolic alterations in patients with AD and tauopathies.

## Materials and methods

### Animals

Tau KO mice (C57BL6/J background) were generated by integrating GFP-encoding cDNA into exon 1 of the mouse MAPT gene (Tucker et al., 2001). In all experiments, only males were used. All animals were maintained in standard animal cages under conventional laboratory conditions (12-h/12-h light/dark cycle, 22°C), with ad libitum access to food and water. The animals were maintained in compliance with European standards for the care and use of laboratory animals and experimental protocols approved by the local Animal Ethical Committee (agreement APAFIS#2264-2015101320441671 from CEEA75, Lille, France).

### Hippocampal slices preparation

Mice were killed by cervical dislocation. Whole brains were rapidly removed from the skull and immersed for 1 min in ice-cold artificial cerebrospinal fluid (ACSF) solution containing (mM): 117 NaCl, 4.7 KCl, 2.5 CaCl_2_, 1.2 MgCl_2_, 1.2 NaH_2_PO_4_, 25 NaHCO_3_, and 10 glucose. The ACSF was continuously oxygenated with 95% O2, 5% CO2 to maintain the proper pH, 7.4. The hippocampi were quickly removed and placed into ice-cold ACSF. Thereafter, 400-µm-thick transverse slices were prepared at 4°C with a chopper (McIlwain Tissue Chopper, TC752). Slices were then placed in a holding chamber containing oxygenated ACSF and kept at room temperature for at least 1 h before processing. Slices from KO tau strain were used either for electrophysiological recordings (see next section) or for biochemical experiments. In the latter case, slices were treated 10 min with 200 nM insulin.

### Electrophysiological recordings

Three to five slices were transferred into a custom-made submerged-type recording chamber and maintained at 32°C constantly superfused with oxygenated ACSF (95% O_2_, 5% CO_2_) at a rate of 2.5 ml/min. After an incubation time of 90 min, fEPSPs were evoked in the stratum radiatum of the CA1 region by electrical stimulation of Schaffer collateral-commissural fibers using ACSF-filled glass micropipettes (2–5 MΩ). For stimulation, 100-µs biphasic constant-current square pulses were generated by an A-M Systems isolated pulse stimulator 2100 and delivered at a frequency of 0.033 Hz using a tungsten electrode with a 50-µm exposed tip. Signals were recorded and amplified with an A-M Systems 1700 differential amplifier (bandpass filtered at 5 Hz and 10 kHz, respectively), digitized using a CED 400 micro AD-converter; Cambridge Electronic Devices), and then further analyzed on-line using custom-made software. To analyze synaptic transmission efficiency, input/output curves were constructed by applying single stimuli in increments of 10 µA from 0 to 90 µA. For subsequent experiments, the intensity of the stimulation was adjusted to elicit a fEPSP of 35% of the maximum and was kept constant throughout the experiment. In all experiments, baseline synaptic transmission was monitored for 30–60 min before drug administration. All values from the onset of insulin application until the end of recording were expressed relative to the control level (percentage of baseline).

### Surgical procedures and injections

Bilateral cannulae (C235G-3.0/SPC with a removable dummy wire; Plastics One) were stereotaxically implanted into lateral ventricle (coordinates with respect to bregma: −0.7 mm anteroposterior, ±1.5 mm mediolateral, −2 mm dorsoventral, according to the Paxinos and Franklin (2013) in anesthetized mice (1.5% isoflurane). Animals were allowed to recover for 1 wk. Over the next 15 d, animals were habituated to the contention and injection procedure. Injections were performed in awake and freely moving mice. Animals were injected with 1 μl (per ventricle) of a solution containing either vehicle (PBS, pH 7.5) or 5 mg/ml insulin at the rate of 0.4 µl/min via cannulae PE50 tubing (Plastics One) connected to a 10-µl Hamilton syringe pump system (KDS310; KD Scientific). The tubing was left in place for another 1 min at the end of each injection and the cannulae capped to prevent reflux of the injected solution. In metabolic cage experiments, icv injection of insulin took place 1 h after light extinction. In experiments where the effects of icv insulin toward IRS-1 and Akt phosphorylations were evaluated, insulin was injected in nonfasted animals and mice sacrificed 1 h after injection. Regarding the in vivo leptin sensitivity measurement, three intraperitoneal injections of 3 mg/kg leptin were made (in 5 mM sodium citrate buffer, pH 4.0) at 18:00 for 3 d, and body weights and food intake were measured as described previously (Coupé et al., 2012).

### Cell culture

N1E-115 mouse neuroblastoma cells were grown in Dulbecco’s modified Eagle’s medium supplemented with 10% fetal calf serum without pyruvate, 2 mM l-glutamine, and 50 U/ml penicillin/streptomycin (Invitrogen) in a 5% CO2 humidified incubator at 37°C. Transfection with plasmid constructs (pcDNA4, empty of containing 1N4R human tau sequence) was performed 24 h after cell seeding into six-well plates (for biochemical studies) using ExGen500 (Euromedex), according to the manufacturer’s instructions, for 48 h. When appropriate, cells were treated for 10 min with 200 nM insulin.

### Biochemical analysis

Proteins from slices were extracted in RIPA buffer (10 mM Tris-HCl, 320 mM sucrose, 150 mM NaCl, NP-40 1%, sodium deoxycholate 0.5%, and SDS 0.1%, pH 8.0) for Western blot analysis from slices. Protein amounts were evaluated using the BCA assay (Thermo Fisher Scientific), subsequently diluted with LDS 2× supplemented with reducing agents (Invitrogen), and then separated on NuPAGE Novex gels (Invitrogen). Proteins were transferred to nitrocellulose membranes, which were then saturated (5% nonfat dry milk or 5% BSA) in Tris 15 mmol/L, pH 8, NaCl 140 mmol/L, and 0.05% Tween and incubated with primary and secondary antibodies. Signals were visualized using chemiluminescence kits (ECL; GE Healthcare) and a LAS3000 imaging system (Fujifilm). Results were normalized to β-actin, and quantifications were performed using ImageJ software (Scion Software). β-Actin or GAPDH served as a loading control. For immunoprecipitation experiments, extraction was realized in a buffer composed of 50 mM Tris-HCl, pH7.4, 150 mM NaCl, 1 mM EDTA, 1% Nonidet, and 0.5% sodium deoxycholate with protease inhibitors and samples centrifuged at 12,000 *g* (10 min, 4°C). 200 μg supernatant protein was incubated with antibodies targeting tau (tau5, AHB00429; Invitrogen) or PTEN (Ab70326; Abcam) and anti-IgG mouse beads (TrueBlot; eBioscience) for one night at 4°C. Unbound fraction was obtained in the supernatant after a centrifugation at 2,000 *g* (5 min) and the eluate recovered in the pellet after three centrifugations at 2,000 *g* (5 min).

Antibodies used for Western blots were as follows. Antibodies against Akt (#9272), pSer473-Akt (#9271), IR β (#3025), pSer616IRS-1 (#3203), pSer636IRS-1 (#2388), PI3K-P85 (#4292), PTEN (#9552), PTP1B (#5311) and SHIP2 (#2730) were from Cell Signaling Technologies. Anti-pY612 IRS-1 was from Thermo Fisher Scientific (44-816G). Anti–IRS-1 was from EMD Millipore (06–248). β-Actin was from Sigma-Aldrich (A5441), GAPDH was from Santa Cruz Biotechnology (FL-1-335). Anti–total tau was a homemade antibody (Cter) recognizing the last 15 amino acids of the C terminus. Anti–tau pSer396, pSer404, and pSer262 were from Invitrogen (44752G, 44758G, and 44750G).

To evaluate the phosphorylation of IR, hippocampal slices from tau KO mice and littermate controls were treated for 10 min with 200 nM insulin and then frozen in liquid nitrogen for subsequent analysis. Proteins were extracted in buffer containing 50 mM Tris, pH 8, 137 mM NaCl, 10% glycerol, 1% NP-40, protease inhibitors (1 µg/ml each: 4-(2-aminoethyl)benzenesulfonyl fluoride hydrochloride, leupeptin, aprotinin, antipain, and pepstatin), and phosphatases inhibitors (50 mM NaF, 10 mM β-glycerophosphate, and 1 mM orthovanadate). Precipitation of the IR on wheat-germ lectin agarose beads (wheat-germ lectin eluate) was performed as described previously (Issad et al., 1995) and analyzed by Western blotting using anti–IR^pY1158-1162-1163^ antibody (Invitrogen) and anti–IRβ antibody (Santa Cruz Biotechnology, Inc.).

### Gene expression analysis

Visceral adipose tissue total RNA was isolated using the guanidinium isothiocyanate phenol/chloroform extraction method. 1 μg total RNA was reverse transcribed to cDNA using the high-capacity cDNA reverse transcription kit (Applied Biosystems) according to the manufacturer’s instructions. Reverse-transcribed cDNAs were quantified by Brilliant III Ultra-Fast SYBR green-based real-time PCR using specific oligonucleotides (cyclophilin: forward, 5′-GCATACGGGTCCTGGCATCTTGTCC-3′; reverse, 3′-ATGGTGATCTTCTTGCTGGTCTTGC-5′; leptin: forward, 5′-GGTGTGAAAGAACCTGAGCTGAGG-3′; reverse, 5′-CAGTGGATGCTAATGTGCCCTG-3′) on a Stratagene Mx3005P (Agilent Technologies) apparatus. mRNA levels were normalized to cyclophilin A expression as an internal control, and mRNA fold induction was calculated using the comparative Ct (2−ΔΔCt) method.

### Cell-free measurements of PTEN activity

In vitro PTEN activity was evaluated in the presence of recombinant 2N4R-tau (obtained as described in Barthélemy et al., 2016) by monitoring the production of phosphatidylinositol 4,5 bisphosphate (PIP2) from phosphatidylinositol 3,4,5 triphosphate (PIP3) by recombinant PTEN. Recombinant PTEN (Berlin Pharma) and tau protein or albumin as a control was incubated 1 h at 37°C in the presence of PIP3 in low-bind 0.5-ml Eppendorf tubes. Then, the samples were exposed to an inactivating temperature of 95°C, and PIP2 levels were determined using ELISA (Echelon-Bioscience) according to the manufacturer’s instructions.

### BiFC plasmids

For the BiFC assay, we generated fusions of our proteins of interest with Venus fluorescent protein fragments as previously described (Outeiro et al., 2008). In particular, we used a larger N-terminal fragment of Venus (VN), corresponding to amino acids 1 to 158, and a smaller C-terminal fragment (VC), corresponding to amino acids 159 to 239. Tau and PTEN cDNA were cloned to the 3′ end of the VN fragment (VN-tau, VN-PTEN) and upstream of the VC fragment (tau-VC, PTEN-VC) by PCR using specific primers, including restriction enzyme sites AflII at the 5′ end and Xho1 at the 3′ end. The primers used were VN-tau: 5′-GCGCTTAAGATGGCTGAGCCCCGCCAGGAGTTCGAAGTGATGG-3′ and 5′-CGCCTCGAGTCACAAACCACCCTGCTTGGCCAGGGAGGCAGAAGACACC-3′; tau-VC: 5′-GCGCTTAAGATGGCTGAGCCCCGCCAGGAGTTCGAAGTGATGG-3′ and 5′-CGCCTCGAGCAAACCCTGCTTGGCCAGGGAGGCAGACACCTCG-3′; VN-PTEN: 5′-GGGCTTAAGATGACAGCCATCATCAAAG-3′ and 5′-CCCCTCGAGTCAGACTTTTGTAATTTGTGTATGC-3; and PTEN-VC: 5′-GGGCTTAAGATGACAGCCATCATCAAAG-3′ and 5′-CCCCTCGAGGACTTTTGTAATTTGTGTATGCTGATC-3′.

PCR fragments were restriction digested and cloned into aSyn BiFC constructs (Outeiro et al., 2008; Gonçalves et al., 2010; Lázaro et al., 2014). HEK 293 cells were grown in Dulbecco’s modified Eagle’s medium (PAN Biotech) supplemented with 10% fetal calf serum and 1% penicillin/streptomycin, at 37°C in 5% CO_2_. The cells were transfected 24 h after plating using Metafectene (Biontex Laboratories) according to the manufacturer’s instructions. 48 h after transfection, cells were washed with PBS and fixed with PBS/4% paraformaldehyde. The cells were permeabilized with PBS/0.1% Triton X-100, blocked for 1 h with 1.5% BSA, and incubated overnight with the primary antibody for tau (HT7, MN1000, 1:1,000; Thermo Fisher Scientific). After three washes with PBS, the secondary antibody anti–mouse IgG conjugated to Alexa Fluor 555 (1:1,000, Thermo Fisher Scientific) was added for 1 h at room temperature. Cell nuclei were stained with Hoechst dye (Hoechst 33258; Molecular Probes). Fluorescent images were captured either using a microscope (DMI 6000B; Leica Biosystems) or an automatic microscope (IX81-ZDC; Olympus). Venus and tau fluorescence was quantified from 16 fields of cell images automatically collected with the Olympus microscope and analyzed by the ScanR software.

### BRET

PIP3 production was monitored as follows. 300,000 N1E115 cells were transfected using lipofectamine 2000 with 700 ng Luc-Akt-PH cDNA (PH domain of Akt is fused to Renilla luciferase), 300 ng pEYFP-Mem cDNA (YFP-targeted to the plasma membrane), and either 500 ng pcDNA3-hTau46 or pcDNA3. Cells were preincubated for 10 min with coelenterazine and then stimulated with 5 nM insulin. PIP3 production was monitored in real time using BRET as described previously (Pierre-Eugene et al., 2012).

### Metabolic cages

Spontaneous feeding and locomotor activity (beam breaks/hour) were measured using metabolic cages (Labmaster; TSE Systems). In brief, food intake and locomotor activity were monitored continuously for 24 h. Food intake was measured by the integration of weighing sensors fixed at the top of the cage from which the food containers were suspended into the home cage. Locomotor activity was assessed using a metal frame placed around the cage. Evenly spaced infrared light beams are emitted along the x axis. Beam interruptions caused by movements of the animals are sensed and registered at high resolution. The sensors for detection of movement operate efficiently under both light and dark phases, allowing continuous recording. Mice were housed individually and acclimated to the home cage for 72 h prior experimental measurements.

### Biochemical plasma parameters

Plasma was collected at the tail vein after a 6-h fasting, and parameters were determined as follows. Blood glucose was measured using the Accu-Chek Performa glucometer (Roche). Plasma insulin and leptin were measured using ultrasensitive insulin ELISA (Mercodia AB) and a mouse/rat leptin enzyme immunoassay kit (Spibio), respectively. Total and high-density lipoprotein (HDL) cholesterol as well as triglycerides were measured by enzymatic method using ready-to-use kits (Biomérieux). Non–HDL cholesterol levels were calculated by subtracting HDL from total cholesterol.

### Glucose tolerance tests

Glucose tolerance was assessed using the intraperitoneal glucose tolerance test after a 6-h fast. 1 g/kg D(+)glucose (Sigma-Aldrich) was injected intraperitoneally. Blood glucose was then measured at 0, 15, 30, 60, 90, and 120 min after injection.

### Assessment of potential association between MAPT haplotypes and glycemic traits in European individuals

The *MAPT* SNP rs1052553 was selected because it is a tag-SNP for the H1 and H2 haplotypes (Donnelly et al., 2010). The A (major) and G (minor) alleles of rs1052553 capture the H1 and H2 haplotypes, respectively. To examine the potential association of rs1052553 with glycemic traits in European subjects, the publicly available GWAS data contributed by the MAGIC (the Meta-Analyses of Glucose and Insulin-related traits Consortium) investigators were downloaded from www.magicinvestigators.org (Saxena et al., 2010; Prokopenko et al., 2014).

### Statistics

Results are expressed as mean ± SEM. Statistical analyses were performed using the Student’s *t* test and one- or two-way ANOVA followed by a post-hoc Fisher’s least significant difference (LSD) test using GraphPad Prism Software. P-values less than 0.05 were considered significant.

### Online supplemental material

Fig. S1 shows additional metabolic indexes for tau KO mice. Fig. S2 shows peripheral metabolic changes in mice heterozygous for tau compared with WT and KO animals. Fig. S3 shows a model of regulation of insulin signaling by tau.

## Supplementary Material

Supplemental Materials (PDF)
