# Supplementary material for: Tau deletion promotes brain insulin resistance
Source: J Exp Med. 2017 Aug 7;214(8):2257–69. doi: 10.1084/jem.20161731 (PMC5551570; doi:10.1084/jem.20161731)
Supplement: Supplemental Materials (PDF) [file JEM_20161731_sm.pdf]

The Journal of Experimental Medicine

SUPPLEMENTAL MATERIAL

Marciniak et al., https://doi.org/10.1084/jem.20161731

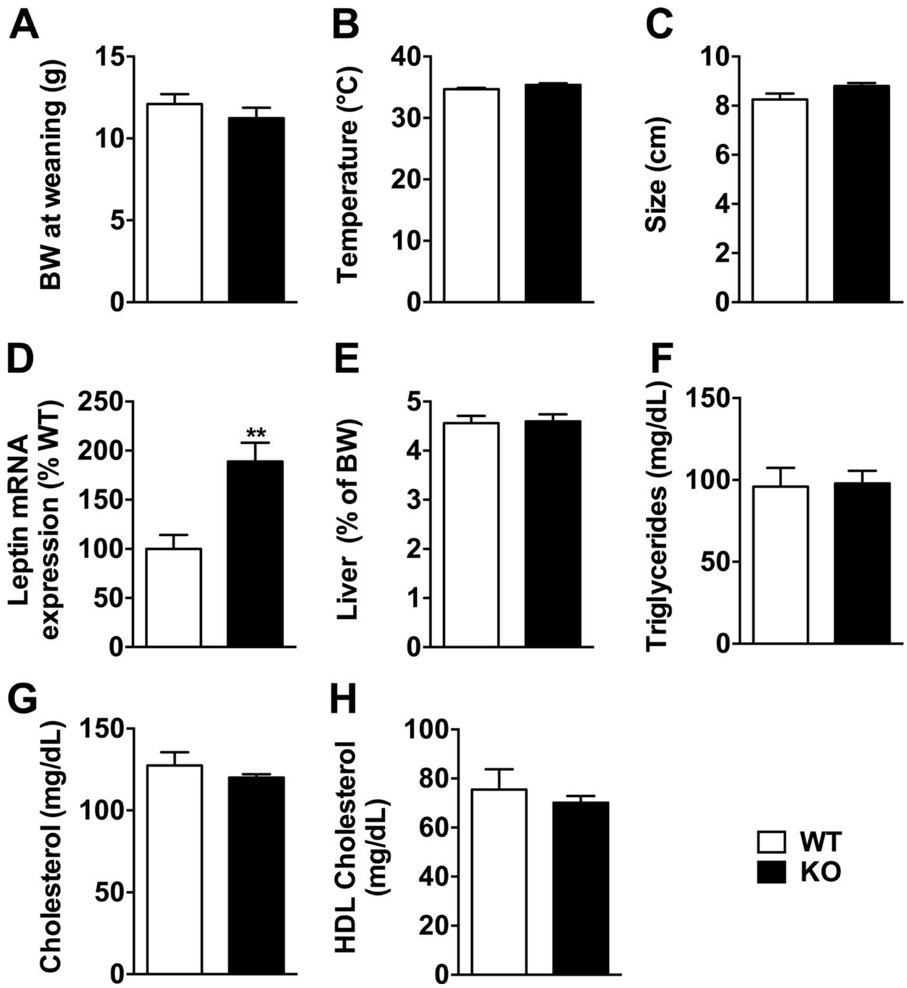

Figure S1. **Additional metabolic indexes for tau KO mice.** (A) Body weight (BW) of animals at weaning. (B) Body temperature. (C) Nose-anus length. (D) Leptin mRNA expression in visceral adipose tissue. (E) Liver weight. (F–H) Plasma triglycerides, total cholesterol, and HDL cholesterol levels. **, $P < 0.001$, Student's *t* test. Littermate controls are indicated as open bar and tau KO animals as a black bars. Mice were 7 mo old at time of sacrifice. Data show mean ± SEM from 8 (A), 5–6 (B), 5–6 (C), 5 (D), 11–14 (E), and 5–10 (F–H) mice per group acquired from one independent experiment.

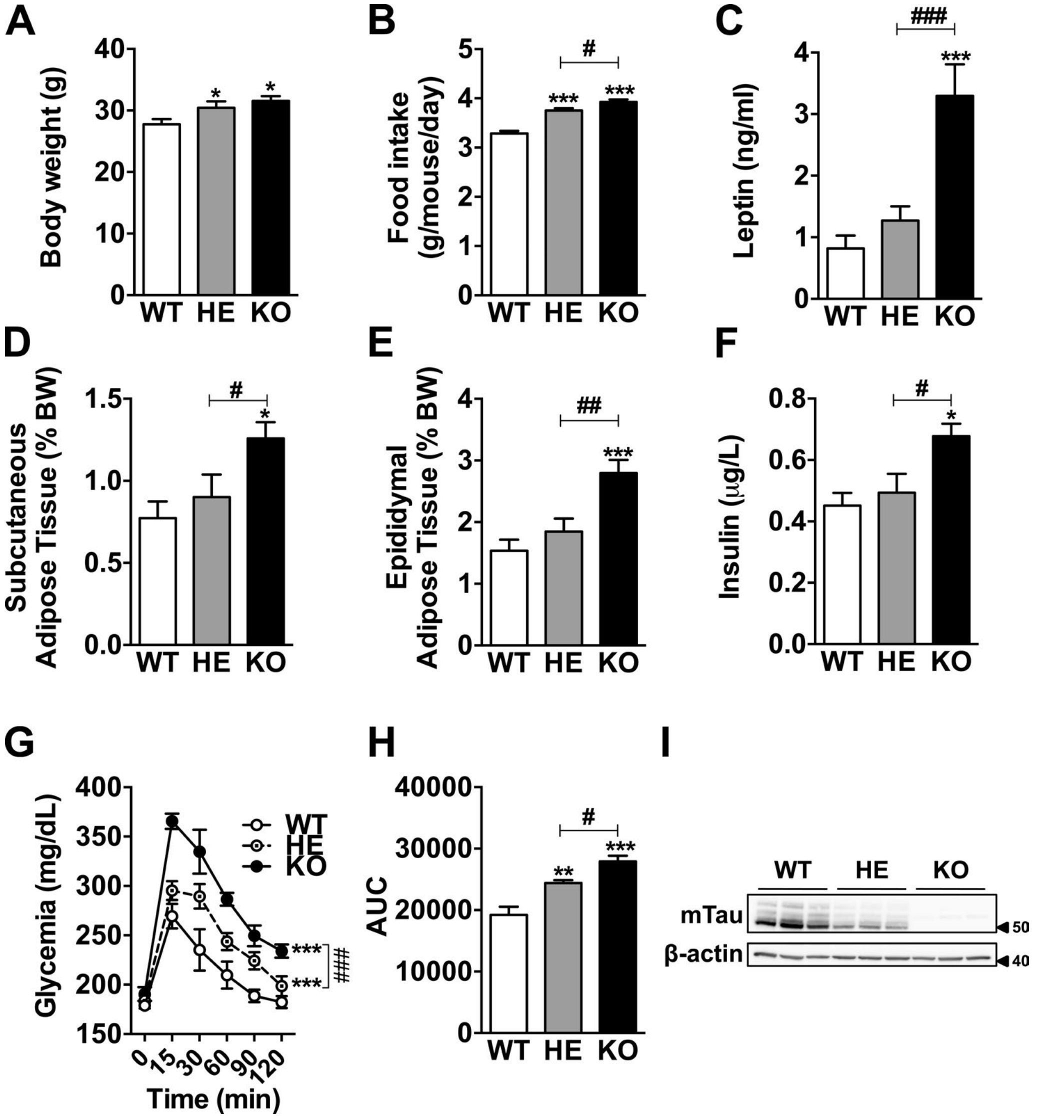

Figure S2. **Peripheral metabolic changes in mice heterozygous for tau compared with WT and KO animals.** (A) Body weight (*, P < 0.05 vs. WT, one-way ANOVA, LSD Fisher's post-hoc test). (B) Food intake (***, P < 0.001 vs. WT; #, P < 0.05 vs. KO, one-way ANOVA, LSD Fisher's post-hoc test). (C) Plasma leptin (***, P < 0.001 vs. WT; ###, P < 0.05 vs. KO, one-way ANOVA, LSD Fisher's post-hoc test). (D and E) Subcutaneous and visceral adipose tissue weights (*, P < 0.05 and ***, P < 0.001 vs. WT; #, P < 0.05 and ##, P < 0.01 vs. KO; one-way ANOVA, LSD Fisher's post-hoc test). (F) Insulinemia (*, P < 0.05 vs. WT; #, P < 0.05 vs. KO, one-way ANOVA, LSD Fisher's post-hoc test). (G and H) Intraperitoneal glucose tolerance test (***, P < 0.001 vs. WT; ###, P < 0.001 vs. KO, two-way ANOVA). For AUC (area under curve; **, P < 0.01 and ***, P < 0.001 vs. WT; #, P < 0.05 vs. KO; two-way ANOVA). Littermate controls are indicated as open circles/bars, tau KO as black circle/bars, and tau heterozygous mice as gray bars/dashed line. Mice (males) were 7 mo old at time of investigation and sacrifice. (I) Brain murine tau expression in WT, HE, and tau KO mice. Molecular mass is indicated in kilodaltons. Data show mean ± SEM from 5–6 (A), 17 (B), 4–5 (C), 5–6 (D and E), 5–9 (F), 6 (G and H), and 3 (I) mice per group acquired from one independent cohort of animals versus results shown in Fig. 3. HE, heterozygous mice.

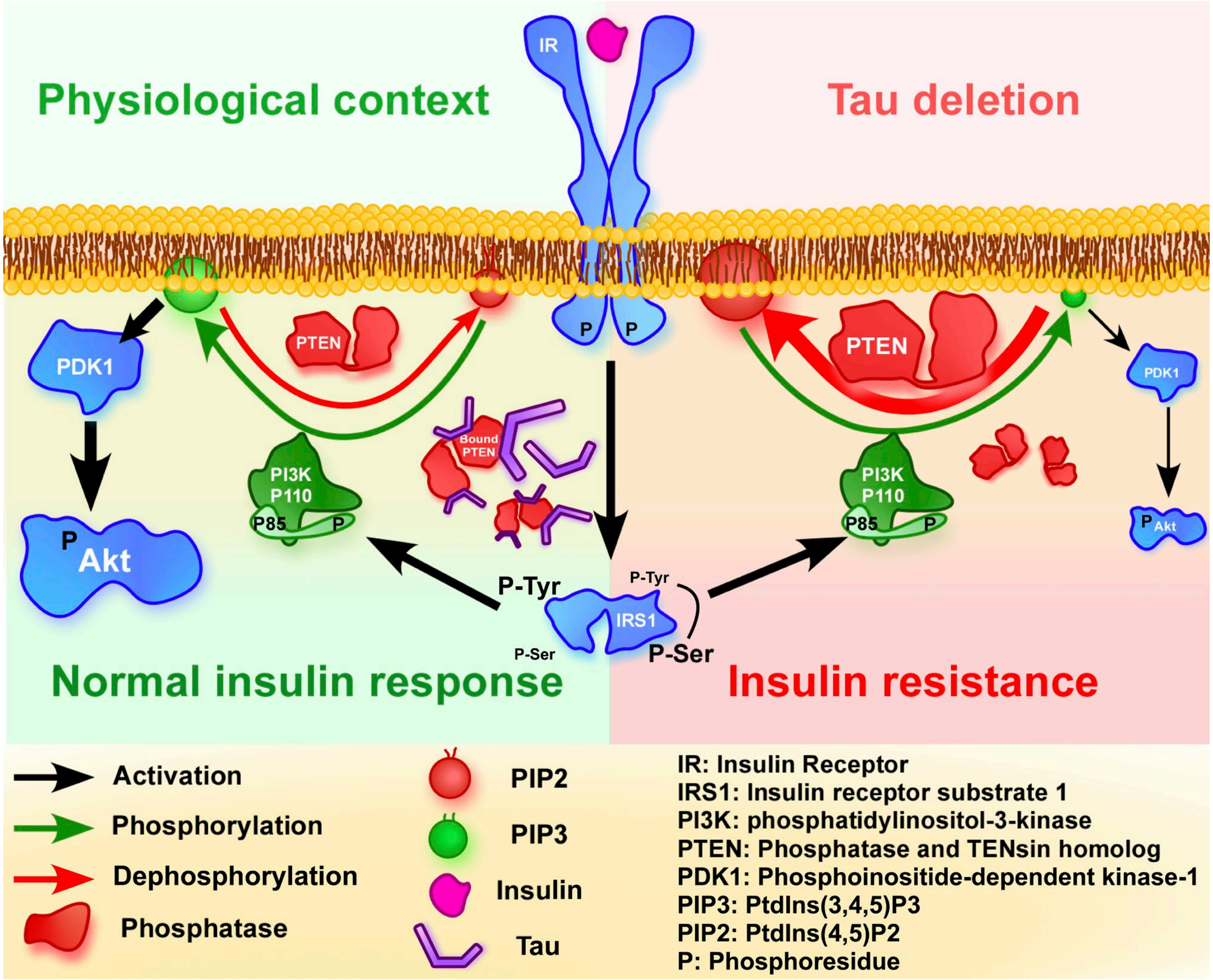

Figure S3. **Model of regulation of insulin signaling by tau.** Binding of insulin to its receptor triggers autophosphorylation at intracellular tyrosine residues, leading to the recruitment of IRS1 and its phosphorylation on tyrosine. The phosphorylation of tyrosine or serine residues of IRS1 activates or inhibits, respectively its signaling ability. Activated IRS1 in turn phosphorylates/activates PI3K. PI3K in turn phosphorylates PtdIns(4,5)P2 to form PtdIns(3,4,5)P3. PtdIns(3,4,5)P3 then activates PDK1, which phosphorylates Akt, transmitting to downstream effectors the insulin signal that favors brain plasticity and energy homeostasis. Formation of PtdIns(3,4,5)P3 is controlled by the lipid phosphatase activity of PTEN. In a physiological context, a fraction of tau protein binds a fraction of PTEN, hence reducing its lipid phosphatase activity and favoring the formation of PtdIns(3,4,5)P3 over PtdIns(4,5)P2. After tau deletion, two mechanisms occur simultaneously. First, IRS1 undergoes phosphorylation on serine residues together with a reduction of tyrosine phosphorylation. IRS1 is thus less activable. Second, the reduction of available tau protein relives the tonic inhibition it exerts on PTEN, enhancing the dephosphorylation rate of PtdIns(3,4,5)P3 and thereby reducing the response to insulin. Both mechanisms participate in insulin resistance induced by loss of tau, which affects memory processes and energy homeostasis in the brain.
